# Relating Ettringite Formation and Rheological Changes during the Initial Cement Hydration: A Comparative Study Applying XRD Analysis, Rheological Measurements and Modeling

**DOI:** 10.3390/ma12182957

**Published:** 2019-09-12

**Authors:** Cordula Jakob, Daniel Jansen, Neven Ukrainczyk, Eddie Koenders, Ursula Pott, Dietmar Stephan, Jürgen Neubauer

**Affiliations:** 1 GeoZentrum Nordbayern, Department of Geography and Geosciences, FAU Erlangen-Nuernberg, 91054 Erlangen, Germany; juergen.neubauer@fau.de; 2 Institute of Construction and Building Materials, TU Darmstadt, 64287 Darmstadt, Germany; ukrainczyk@wib.tu-darmstadt.de (N.U.); koenders@wib.tu-darmstadt.de (E.K.); 3 Building Materials and Construction Chemistry, TU Berlin, 10623 Berlin, Germany; u.pott@tu-berlin.de (U.P.); stephan@tu-berlin.de (D.S.)

**Keywords:** hydration kinetics, rheology, ettringite formation, OPC, temperature, modeling, scanning electron microscopy (SEM)

## Abstract

In order to gain a deeper understanding of the rheological development of hydrating ordinary Portland cement (OPC) pastes at initial state, and to better understand their underlying processes, quantitative X-ray diffraction (XRD) analysis and rheological measurements were conducted and their results combined. The time-dependent relation between phase development and flow behavior of cement paste was investigated at two different temperatures (20 and 30 °C), over a period of two hours. Regarding the phase development during hydration, ettringite precipitation was identified as the dominant reaction in the first two hours. For both temperatures, the increasing ettringite content turned out to correlate very well with the loss of workability of the reacting cement paste. An exponential relationship between ettringite growth and flow behavior was observed that could be explained by applying the Krieger-Dougherty equation, which describes the influence of solid fraction on the viscosity of a suspension.

## 1. Introduction

The workability of fresh cementitious mixtures is of great interest for the construction industry as well as for academia. New technologies, like 3D-printing and the construction of supertall buildings, put special requirements on the performance and longleivity of cementitious systems [1,2,3]. For this, it is necessary to enhance and optimize the workability uniquely and according to its application. To accomplish these challenging demands for special concrete mix designs, a deeper understanding of the underlying processes that take place during early cement hydration is necessary, as it strongly influences the rheology/workability of cementitious systems.

The early cement hydration of ordinary Portland cement (OPC) is primarily controlled by two chemical reactions: (1) The silicate and (2) the aluminate reaction, which run simultaneously. The silicate reaction can be represented schematically by the following equation:

alite + water → C–S–H + portlandite(1)

The dissolution of alite leads to the precipitation of poorly-crystalline calcium silicate hydrate (C–S–H) and portlandite [4] (p. 113). Thereby, the nanostructure of C–S–H is changing during hydration from X-ray amorphous, consisting of monomeric silicon tetrahedrons, to a “long-range ordered” dimeric C–S–H, which can be detected by X-ray diffraction (XRD) [5].

During the aluminate reaction ettringite is formed by the reaction of C_3_A with water in the presence of sulfate carriers, mainly anhydrite, bassanite and gypsum, according to the following equation [4] (pp. 182–183):

C_3_A + calcium sulfate + water → ettringite (2)

This complex system of various dissolving cement phases and precipitating hydrate phases is still not yet fully understood and is, therefore, still considered in many scientific studies. Some reviews summarized the state of the art on this particular topic and reported the unsolved questions in the last few years [6,7]. 

During initial hydration, the rheological parameters—yield stress and plastic viscosity—increase [8] (p. 96). This change in flow behavior commences slowly and then proceeds more rapidly [9,10,11]. In literature, there is a general agreement that the rapid solidification at later times corresponds to the setting of the cement paste and is mainly controlled by C–S–H formation [10,11,12,13,14]. This change of the paste behavior can be attributed to the transformation of a suspension into a mechanically interconnected network of particles during the acceleration period of the silicate reaction [15]. In pure alite systems, Nonat [15] stated that this mechanism proceeds when the first hydrates are formed, which solidify the contact points between particles. Portlandite is hereby not the determining phase, but merely C–S–H [15].

However, which particular chemical reaction is controlling the flow behavior in the first period of hydration (commonly the first 2 h) is still under debate. Roussel et al. [16] suggest that from the beginning of hydration on the rheology is primarily influenced by the silicate reaction, in particular by the precipitation of C–S–H. As already described by Gauffinet-Garrault [14], the initial stiffening of the cement paste is explained by the bridging effect of C–S–H, which leads to flocculation. However, Jansen et al. [17] could not find any evidence for an amorphous hydrate phase forming in the first hours of OPC hydration. As well, also, Nonat [15] stated that in pure alite systems there is practically no precipitation of hydrates in the initial period before setting. This is merely the nucleation period of C–S–H. Nevertheless, he found out that already in this period, alite particles agglomerate. This coagulation is caused by attractive forces and is reversible.

Other authors argue that ettringite may be the crucial hydrating phase controlling the rheology of the paste at the beginning of reaction. Gołaszewski [18] found out that the C_3_A content—as the critical phase for the aluminate reaction of the dry cement—has a significant influence on the rheological parameters of mortar. Furthermore, Uchikawa et al. [10] compared cements with different C_3_A contents concerning their ettringite precipitation and yield stress development during hydration. They observed that for higher C_3_A contents in cement, the amount of ettringite formed during hydration and the yield stress, increased as well. Their conclusion was therefore, that ettringite is the main responsible phase for changes in flow behavior of cementitious systems, which is also supported by Winnefeld et al. [19]. They investigated the interaction between polycarboxylate-ether based superplasticizers (PCE) and cement clinker as well as hydrate phases. By comparing adsorption, zeta potential, and rheological measurements, they concluded that the formation and dispersion of ettringite are dominating the workability in fresh cement pastes.

However, not only the hydration time decreases the workability of cement paste, also the ambient temperature is an important factor, which influences the hydration kinetics as well as the rheological properties. Therefore, hot weather environments can interfere the workability and may cause problemes during concrete transport and placement [20]. At higher temperatures, the accelerated dissolution of clinker phases leads to a faster formation of hydrate phases, which results in an increased reaction rate of the cementitious system [21]. This acceleration of the hydration affects the rheological properties, which are also commonly known as strongly temperature-dependent. This was also confirmed by Martini et al. [22], who observed an increase of yield stress and plastic viscosity with increasing ambient temperature.

In the early stage of hydration, cement paste is a suspension consisting of cement phases, hydrate phases, and water (including air). The flow behavior of a suspension is strongly influenced by the volume concentration of particles and their interactions, which can be modeled by applying the Krieger-Dougherty equation [23]. It is defined as follows:

(3) ηηc=(1−ΦΦM)−[η]ΦM

η=apparent viscosity of the suspension$\eta_{c}=\mathrm{apparent}\;\mathrm{viscosity}\;\mathrm{of}\;\mathrm{the}\;\mathrm{liquid}\;\mathrm{phase}$Φ=concentration of solid fraction$\Phi_{M}=\mathrm{maximum}\;\mathrm{of}\;\mathrm{solids}\;\mathrm{concentration}$[η]=intrinsic viscosity of the suspension

where the concentration of the solid fraction Φ is in percent by volume. Thereby the maximum volume fraction $\Phi_{M}$ depends on particle size distribution, particle shape and shear rate. Struble and Sun [24] modeled the viscosity of Portland cement pastes as a function of its concentration, finding the values for $\Phi_{M}$ to be between 0.64 and 0.8. The intrinsic viscosity [η] is a measure of the effect of individual particles on viscosity, and also depends on the particle shape and shear rate and ranges from 4.5 to 6.8 in cement pastes [24]. Although the Krieger-Dougherty equation was derived for dispersed particles, it turned out to be also applicable for flocculated suspensions. Flocculation showed a substantial effect only on the calibrated $\Phi_{M}$ Krieger-Dougherty parameter [24], which shifted the viscosity-concentration curve to the left, making viscosities much higher for same volume fraction.

In order to gain a more significant insight in the underlying processes responsible for the development of the rheological behavior of hydrating cement pastes, in-situ X-ray diffraction (XRD), heat flow calorimetry and rheological measurements were performed. Additionally, Scanning Electron Microscope (SEM) images were taken and the development of the flow behavior of cement paste was modeled by applying the Krieger-Dougherty equation [23]. In the present paper we would like to identify the relevant processes responsible for changes in rheological behaviour of fresh cement pastes.

## 2. Materials and Methods 

An ordinary Portland cement (OPC) of type 42.5 R was used for this study. The cement was provided within the SPP 2005 program of the DFG (Deutsche Forschungsgemeinschaft) “Opus Fluidum Futurum—Rheology of reactive, multiscale, multiphase construction materials” [25]. The characterization of the cement will be available in an article which will be published soon [26]. The quantitative composition of the applied cement is shown in Table 1. The chemical composition was determined by X-ray fluorescence analysis (XRF), and the phase content was characterized applying X-ray diffraction (XRD) combined with Rietveld refinement and the external standard method [27,28].

The cement and the deionized water were equilibrated at the experimental temperatures before mixing. Two temperatures were used for the experiments: 20.0 ± 0.5 °C and 30.0 ± 0.5 °C. The mixing was performed in a KitchenAid 5K45 mixer, and started with 58 rounds per minute (rpm) for 15 s. Then the speed was raised to 125 rpm for 45 s, and after that to 220 rpm for 30 s. This was followed by a 90 s mixing break during which the sides of the bowl were scraped off. In the following 60 s the cement paste was remixed at 220 rpm. For all experiments except the heat flow calorimetry, 650 g cement, and 234 g deionized water (w/c = 0.36) were mixed. For each experimental setup, three measurements were performed for both temperatures.

The heat flow of the cement pastes was recorded with a TAM Air calorimeter. In order to measure meaningful values during the first hour, a custom made internal mixing tool [29] was used, which guarantees artefact-free detection of the early heat evolution. This experimental setup allows equilibration, injection of water, and mixing inside the calorimeter. One g cement and 0.36 g water were mixed at 860 rpm for 1 min. According to the time resolution of the calorimeter, the raw data were corrected by the Tian equation [30]:

(4) $$P_{c}\left(t\right)=P_{0}\left(t\right)+\tau \frac{dP}{dt}$$

$P_{c}=\mathrm{corrected}\;\mathrm{thermal}\;\mathrm{power}\;\left[W\right]$$P_{0}=\mathrm{measured}\;\mathrm{thermal}\;\mathrm{power}\;\left[W\right]$τ=time constant [s]t=time [s]

The phase development of the cement hydration in the first five hours of hydration was determined by in-situ XRD measurements. In order to ensure a constant experimental temperature (20.0 ± 0.2 °C and 30.0 ± 0.2 °C) during the whole timeframe, the cement paste was transferred into a custom made heating and cooling device. By covering the paste with a 7.5 µm thick Kapton^®^ polyimide film water evaporation and CO_2_ intake were avoided. Every 10 min a diffraction pattern was recorded by a diffractometer (Bruker AXS D8 Advance) in Bragg-Brentano geometry. A step width of 0.0236° 2θ was employed, and the resulting patterns ranged from 7° to 55° 2θ. The diffraction patterns were refined by the Rietveld method using the software TOPAS 5.0 (Bruker AXS) [27]. Additionally, the external standard G-Factor method [28] was applied, which enables an absolute quantification of the phases present in the paste, independent of the amount of amorphous phases present.

**Figure 1:**
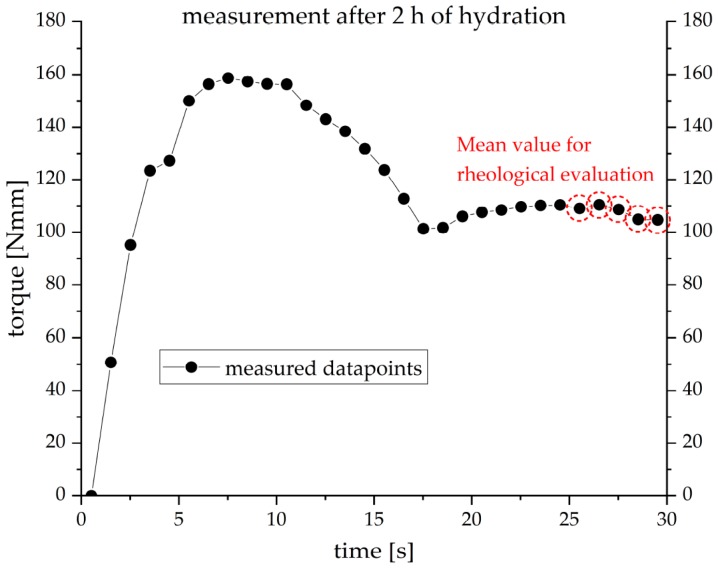
Exemplary result of a rheological measurement after two hours of hydration at a speed of 2 rpm at 20 °C (w/c = 0.36).

In order to determine the rheological development of cement paste, rheological measurements were performed by a speed-controlled rotational rheometer (Schleibinger Viskomat NT). The torque was measured by using a fishbone paddle geometry at a velocity of 2 rpm. The advantage of this geometry is that the surface of the tool is small and thus it is possible to measure the cement paste rheology during ongoing hydration process for a long time after water addition. For this geometry it is not possible to calculate the viscosity nor the yield stress. Therefore, here the rheological properties are expressed by the generated torque. The measuring range of the torque is between 0 and 500 N·m, and the angle accuracy of the rheometer is 0.02 degrees. To avoid destruction of the freshly formed hydrading phases, and its effect on the rheology of cement paste, a low velocity of 2 rpm was applied. A new sample was prepared for each measurement and stored at the designated experimental temperature until testing. Pre-shear was waived to avoid the destruction of the first forming crystals. A velocity of 2 rpm was applied for 30 s, and meanwhile, every second one data point was recorded. A plot of a measurement performed after two hours of hydration at a speed of 2 rpm is shown in Figure 1. The mean value, which was used for our results, was calculated from the last five data points. 

For SEM investigations the cement hydration was stopped after three defined times of hydration (15 min, 2 h and 4 h) by an exchange of the water with isopropanol. This exchange was done three times. After that, the sample was dried by freeze drying. The dried powder was analysed by a Scanning Electron Microscope (Zeiss GeminiSEM 500 NanoVP, Jena, Germany). Before the measurement, the sample was covered by a 4.5 nm thick gold layer. For the images a secondary electron (SE) detector in a high vacuum was used and the electrons were accelerated in an electric field with a voltage of 15 kV.

The equation of Krieger and Dougherty [23] considers the solid fraction of a suspension in percent by volume (vol. %). Therefore, the quantitative phase content analyzed by in-situ XRD in percent by weight (wt. %) has to be converted into values in vol. %. This was done by dividing the content in wt. % (g/100 g) of every single phase by the density of this phase. The following densities of the phases are used: alite 3.17 g/cm^3^; belite 3.30 g/cm^3^; anhydrite 2.93 g/cm^3^; bassanite 2.73 g/cm^3^; quartz 2.65 g/cm^3^; brownmillerite 3.74 g/cm^3^; C_3_A 3.04 g/cm^3^; periclase 3.58 g/cm^3^; calcite 2.71 g/cm^3^; gypsum 2.30 g/cm^3^; ettringite 1.80 g/cm^3^; water 1.00 g/cm^3^. With the resulting volume of each phase in cm^3^/100g the whole volume of the cement paste and consequently, the vol. % of every single phase could be calculated effectively. The solid fraction of the cement paste is then the sum of all solid phases (non-dissolved cement phases and precipitated hydrate phases).

Regression and interpolation analysis was performed using the Levenberg–Marquardt method of optimization in software Origin Pro. The solid phase content of the experimental data was interpolated to the hydration time values corresponding to torque measurements, using a (Hill) growth function (resulting in R^2^ = 0.9999) to fit the evolution of solid-phase content in the experimental data.

## 3. Results

**Figure 2:**
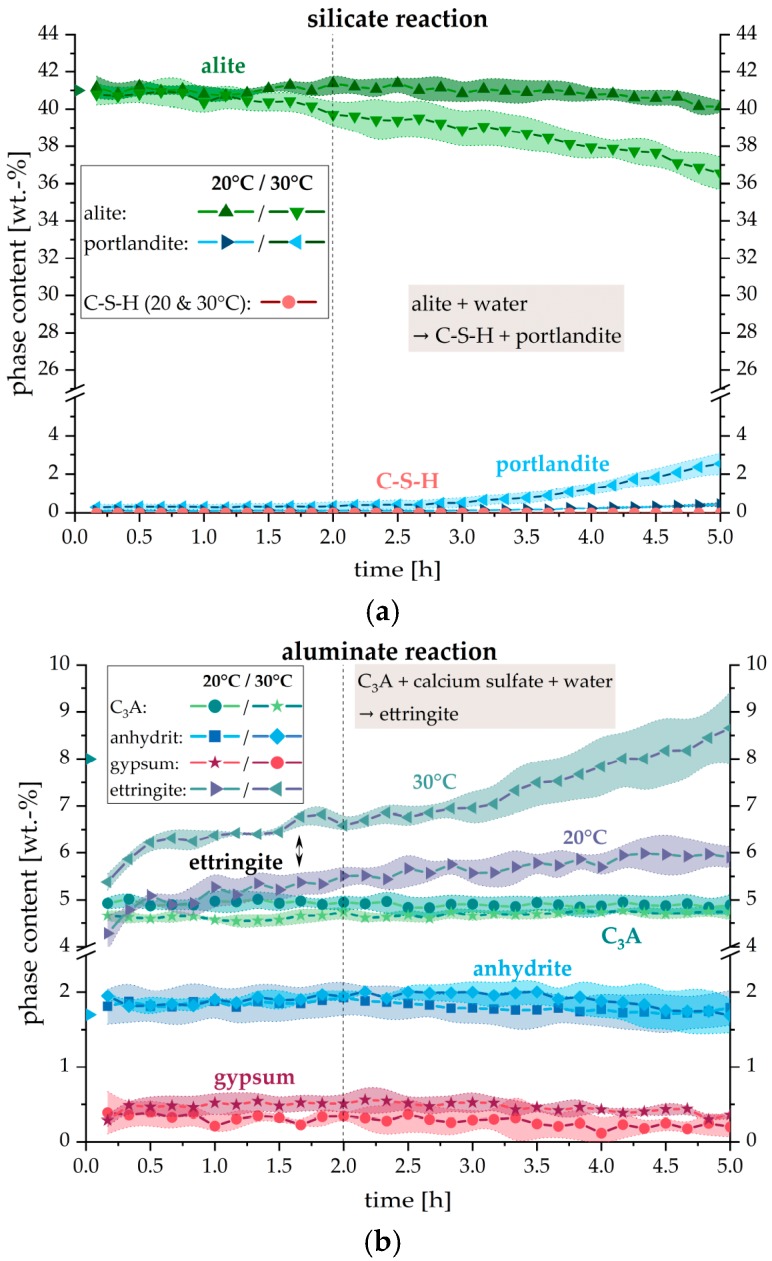
Phase development with respect to (**a**) the silicate and (**b**) the aluminate reaction of CEM I during hydration within five hours after contact with water at 20 °C and 30 °C (w/c = 0.36). Colored background indicates the error of 3 independent measurements. Expected values at the time of mixing: alite = 41.0 wt. %, C_3_A = 8.0 wt. % and anhydrite = 1.7 wt. % (triangles at t = 0 h).

Figure 2 shows the quantitative evolution of the phase contents as determined by in-situ XRD measurements. Since an external standard method was used, the wt. % given are absolute values of all phases considering the significant amorphous content caused by the water added for hydration and also considering not determined phases. It can be seen from Figure 2 that there is a significant difference between the reaction at 20 °C and 30 °C. Within the first hours of hydration (until ~2 h after the addition of water), which is actually the most interesting period for concrete pumping and digital fabrication, the actual phase development of most constituents does not differ between both temperatures. However, solely the precipitation of ettringite shows considerable quantitative differences. Within the first two hours, no evidence of a significant alite dissolution as well as no portlandite and no C–S–H precipitation emerged from the measurements. 

It should be taken into account that gypsum is not present in the dry cement CEM I 42.5 R and is thus a hydrate phase which precipitates during the first minutes of hydration (before the first pattern was recorded). The highly soluble sulfate carriers like bassanite and arcanite are totally dissolved within the first 10 min and are therefore not shown in the figure.

**Figure 3:**
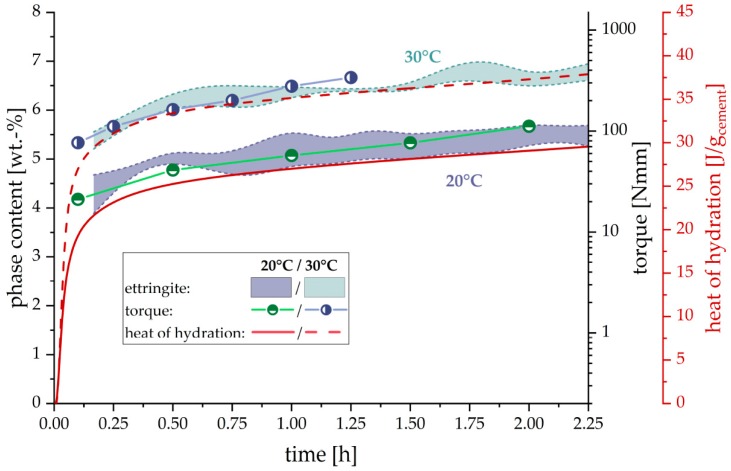
Comparison of ettringite content, rheological properties and heat of hydration during the first two hours of hydration at 20 °C and 30 °C (w/c = 0.36). Note that the phase content and the heat of hydration are linearly scaled whereas the torque is scaled logarithmically. Ettringite content is displayed as colored background with respect to the 3 independent measurements.

Figure 3 focuses on the quantitative development of ettringite precipitation for the two different temperatures and are compared with the evolution of heat release and measured torque of the cement paste over a period of two hours after water addition. With increasing ettringite content the heat of hydration as well as the measured torque increases as well. The higher ettringite content at 30 °C, measured over the two hours, compared to the 20 °C results, correlate very well with the increased heat release and torque. 

**Figure 4:**
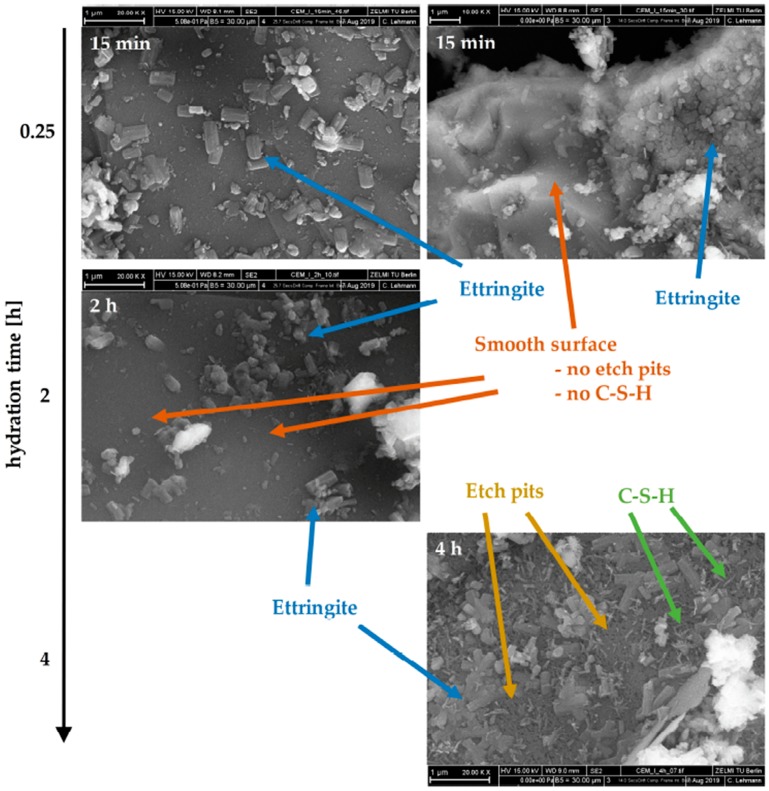
SEM-images of the cement paste at different hydration times (15 min, 2 h and 4 h). For hydration the CEM I was used with a w/c-ratio of 0.36. The precipitation of different hydrate phases can be observed. From the first 15 min of hydration on ettringite precipitates, and at later times C–S–H are visible.

In Figure 4 scanning electron microscope (SEM) images of the cement paste are shown for different hydration times (15 min, 2 h and 4 h). Already after 15 min, a high amount of precipitated ettringite crystals, covering the cement grain surface, could be observed. However, the ettringite crystals are not evenly distributed over the surface of the cement particles. Some areas are covered exensively by many crystals, whereas others are nearly clean. Within the first two hours, clinker surfaces devoid of ettringite precipitates are smooth without any evidence for dissolution of alite (no formation of surface alterations/etch pits) and/or precipitation of portlandite or C–S–H. Only after four hours, indications of alite dissolutions, and distinct C–S–H precipitations could be observed.

**Figure 5:**
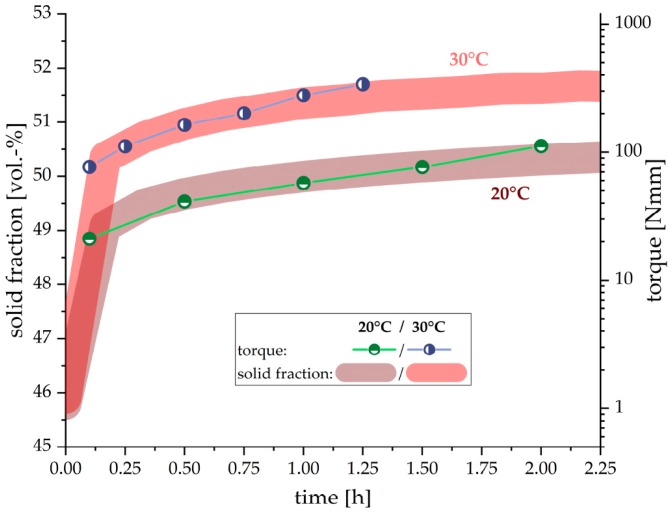
Comparison of the solid fraction of the cement paste (cement & hydrate phases) and the measured torque within the first two hours of hydration at 20 °C and 30 °C (w/c = 0.36). Note that the phase content is linearly scaled whereas the torque is scaled logarithmically. Colored background for solid fraction indicates error margin.

Figure 5 shows the evolution of solid fraction (sum of residual cement phases and hydrate phases) in vol. %, calculated from the results of in-situ XRD measurements within the first two hours. This is compared with results achieved from the rheological measurements. The rising solid fraction in the paste correlates very well with the logarithmic increase of the torque. The measurements at 30 °C shows higher values for solid fraction of the paste as well as the measured torque. The increase compared to the 20 °C turned out to be very consistent over the full duration of two hours.

## 4. Discussion

By comparing the phase evolution of cement paste at initial reaction, analyzed by in-situ XRD measurements and the external standard method (G-factor), with the development of the measured torque at two different temperatures (20 °C and 30 °C) during the first two hours of hydration, the influence of hydrate phases on the workability of the cement paste could be quantified. As shown in Figure 2, ettringite and gypsum are the only detectable hydrate phases in the fresh cement paste. The SEM images in Figure 4 support these findings, where no indication of a significant dissolution of alite and precipitation of C–S–H or portlandite could be found in the considered time period. This is in line with other research [15,17], where no considerable ongoing silicate reaction was observed during the first hours of hydration. The high amount of precipitated ettringite on the cement grain surfaces and the rare occurrence of gypsum crystals confirm the results of the XRD measurements concerning these phases.

Gypsum precipitation during cement hydration indicates an over-sulfated cement and could cause undesirable rapid stiffening of the paste (false set) [4] (pp. 218–219). However, in this case, the amount of gypsum formed is relatively low (below 0.5 wt. %) which minimizes this influence. Furthermore, the gypsum content is nearly identical for both temperatures and not changing during the first hours of hydration. Therefore, it can be assumed that its influence on the rheological properties is low and only limited to a slight initial increase of the measured torque.

By contrast, the ettringite content is constantly increasing during hydration and is clearly influenced by temperature. The excellent correlation in Figure 3 between cumulated heat release of the cement paste and the amount of ettringite precipitated during hydration for both temperatures indicates that the degree of hydration, viz. heat of hydration, is mainly induced by the precipitation of this particular phase. Therefore, it can be inferred that the change of ettringite content might be the parameter responsible for the change in flow behavior of fresh cement pastes.

Furthermore, the ettringite content in cement paste also correlates with the increased torque as measured by the rheometer, evident shown in Figure 3. Also, the increased amount of ettringite precipitated correlates with the higher torque values at 30 °C compared to the samples hydrated at 20 °C. As already stated by Uchikawa et al. [10], it can be assumed that ettringite is the crucial hydrate phase controlling the rheological properties. This can be explained by the high amount of water incorporation into the ettringite structure (45.9 wt. % H_2_O in ettringite) and additional surface area evolved due to the precipitation of the hydrate phase. The high water consumption by ettringite precipitation strongly influences the water/solid ratio in the hydrating cement paste. Therefore, it can be observed from Figure 5, that the solid fraction, calculated from the results of the in-situ XRD measurements, also correlates well with the measured torque values.

**Figure 6:**
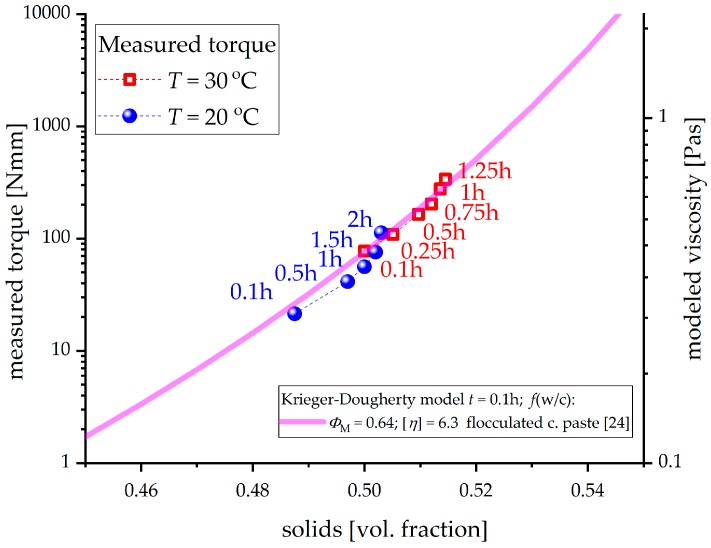
Correlation between measured torque and solids content. To get good agreement between measured and modeled results, only the torque values are scaled to modeled viscosity while keeping the Krieger-Dougherty parameters fixed to the literature values calibrated for the flocculated cement paste (as a function of w/c ratio [24]).

It should be taken into account that the results of the rheological measurements have to be scaled logarithmically to achieve a good correlation with the ettringite content, heat release and solid fraction of the paste, which are scaled linearly. Therefore, it can be assumed that the effect of ettringite precipitation on the rheological properties of the cement paste is of exponential order. With increasing ettringite content during hydration, the effect on the flow behavior becomes much larger. The ongoing water consumption of precipitating ettringite increases the solid fraction in the cement suspension and thus intensifys the interaction between particles. This exponential influence with increasing ettringite content can be modeled by applying the Krieger-Dougerthy equation [23]. Due to the lack of viscosity data, which were impossible to calculate with the applied geometry in this research, the measured torque to modeled viscosity relationship could not be defined. Nevertheless, this model could be used to explain the exponential effect of ettringite formation, which results in an increasing solid fraction, on the rheological properties of the paste, as shown in Figure 6. 

The model parameters are taken from [24], which were already calibrated to experimental results of flocculated cement (CEM I) paste measured only after *t* ≈ 0.1 h, but as a function of w/c, i.e. considering only an initial cement volume fraction of 0.35–0.50. In this paper, the initial solid volume fraction of cement is fixed to 0.45 (calculated according to the applied w/c ratio), and the rheological measurements are performed as a function of hydration time. In contrast to [24], in this research, pastes were not pre-sheared in order not to destroy the flocculated structure of cement and ettringite precipitates. Therefore, the initial viscosity at time *t* ≈ 0.1 h (0.27 Pa s) is scaled to be higher (following the calibrated Krieger-Dougherty model) than the model viscosity from [24] (0.12 Pa s) corresponding to the same cement volume fraction of 0.45. This can be expected according to shear thinning (and thixotropy) behavior of the flocculated suspensions due to the breaking of bonds between individual flocs. Note that in this research, the solid volume fraction comprises residual cement phases as well as precipitated ettringite and gypsum. Therefore, with hydration time, the measured data points in Figure 6 are shifting to higher solid volume fractions. Good correlation between measured torque and solids content was obtained (Figure 6) by adjusting (scaling) only the torque values to the modeled viscosity while keeping the Krieger-Dougherty parameters fixed to the literature values calibrated for the flocculated cement paste (as a function of w/c ratio [24]). Further research is needed to determine whether the model parameters could be estimated from the particle size distribution and particle shape. However, as no direct quantitative relationships could have been established between measured torque and modeled viscosity, the observed correlation presents a first explanation of the exponential influence of ettringite precipitation on the flow behavior of the cement paste during initial cement hydration. Additionally, the influence of changes in the ambient temperature on the rheological properties could be explained as well. The acceleration of the hydration processes at higher temperatures leads to a higher solid content at any point in time during initial hydration, caused by the increased ettringite precipitation.

## 5. Conclusions

State-of-the-art quantitative Rietveld values combined with the external standard method (G-factor) were used to gain more insight into the underlying mechanism that determines the rheological properties of fresh OPC paste. From this the following conclusions could be drawn:

- The amount of ettringite formation strongly depends on temperature and is the main process that drives the change in rheology during initial hydration of cement paste before setting. With increasing ambient temperature the initial ettringite formation is accelerated, which results in higher measured torque values. No other phase in the cement paste could be identified in the initial state to be affected by ambient temperature changes;
- The effect of ettringite precipitation on the rheological properties of cement paste is exponential with hydration time. With increasing ettringite content in the cement paste the effect of additionally formed ettringite on the workability of the cement paste is increased. The high water consumption and the formation of (low density) ettringite changes the water/solid ratio of the paste significantly and, thus, strongly controls the flow behavior of paste. This effect can also be observed from the hydration at different temperatures, as with higher ettringite contents at 30 °C, the measured torque increases faster with the formation of comparable amounts of ettringite than for the measurements at 20 °C;
- The exponential increase of the measured torque of cement paste during initial hydration was correlated by applying the Krieger-Dougherty equation. The correlation between measured torque and modeled viscosity was scaled, but provides a first indication for a logarithmic dependency between the torque and the ettringite content;
- Considerable increase in the solid volume fraction of the cement paste (+4.5 and +5.6 vol. % ettringite) during hydration (within 2.0 h at 20 °C and 1.25 at 30 °C, respectively), is correlated to the increase of the modeled viscosity.

## Figures and Tables

**Table 1:** Chemical (XRF) and mineralogical composition (XRD) of the CEM I 42.5 R.

Oxide	wt. %	Phase	wt. %
CaO	64.4	alite	55.8
SiO_2_	20.4	belite	14.6
Al_2_O_3_	5.4	C_3_A (orthorhombic)	3.6
Fe_2_O_3_	2.6	C_3_A (cubic)	7.3
MgO	1.4	C_4_AF	7.4
K_2_O	0.8	anhydrite	2.3
Na_2_O	0.2	bassanite	2.7
TiO_2_	0.3	arcanite	0.5
P_2_O_5_	0.1	calcite	3.7
Mn_2_O_3_	0.1	quartz	0.9
SO_3_	3.1	periclase	0.4
LOI	1.9		
sum	100.1	sum	99.5
